# Sex-specific genetic modifiers identified susceptibility of cold stored red blood cells to osmotic hemolysis

**DOI:** 10.1186/s12864-022-08461-4

**Published:** 2022-03-23

**Authors:** Fang Fang, Kelsey Hazegh, Alan E. Mast, Darrell J. Triulzi, Bryan R. Spencer, Mark T. Gladwin, Michael P. Busch, Tamir Kanias, Grier P. Page

**Affiliations:** 1 GenOmics, Bioinformatics, and Translational Research Center, RTI International, Research Triangle Park, Durham, NC USA; 2 Vitalant Research Institute, Denver, CO USA; 3 Versiti Blood Research Institute, Blood Center of Wisconsin, Milwaukee, WI USA; 4 Department of Cell Biology, Neurobiology and Anatomy, Medical College of Wisconsin, Milwaukee, WI USA; 5 Department of Pathology, University of Pittsburgh, Pittsburgh, PA USA; 6 American Red Cross, Dedham, MA USA; 7 Pittsburgh Heart, Lung, and Blood Vascular Medicine Institute, University of Pittsburgh, Pittsburgh, PA USA; 8 Division of Pulmonary, Allergy and Critical Care Medicine, Department of Medicine, University of Pittsburgh Medical Center, Pittsburgh, PA USA; 9 Vitalant Research Institute, San Francisco, CA USA; 10 Department of Laboratory Medicine, UCSF, San Francisco, CA USA; 11 Department of Pathology, University of Colorado Denver Anschutz Medical Campus, Aurora, CO USA; 12 Division of Biostatistics and Epidemiology, RTI International, GA Atlanta, USA

**Keywords:** Red blood cell susceptibility to hemolysis, Genome-wide association study (GWAS), Sex difference, NHLBI recipient epidemiology donor evaluation study (REDS)-III—red blood cell omics (RBC-Omics) study, Sex-interaction, Red blood cells, Blood osmotic hemolysis

## Abstract

**Background:**

Genetic variants have been found to influence red blood cell (RBC) susceptibility to hemolytic stress and affect transfusion outcomes and the severity of blood diseases. Males have a higher susceptibility to hemolysis than females, but little is known about the genetic mechanism contributing to the difference.

**Results:**

To investigate the sex differences in RBC susceptibility to hemolysis, we conducted a sex-stratified genome-wide association study and a genome-wide gene-by-sex interaction scan in a multi-ethnic dataset with 12,231 blood donors who have in vitro osmotic hemolysis measurements during routine blood storage. The estimated SNP-based heritability for osmotic hemolysis was found to be significantly higher in males than in females (0.46 vs. 0.41). We identified SNPs associated with sex-specific susceptibility to osmotic hemolysis in five loci (*SPTA1*, *KCNA6*, *SLC4A1*, *SUMO1P1,* and *PAX8*) that impact RBC function and hemolysis.

**Conclusion:**

Our study established a best practice to identify sex-specific genetic modifiers for sexually dimorphic traits in datasets with mixed ancestries, providing evidence of different genetic regulations of RBC susceptibility to hemolysis between sexes. These and other variants may help explain observed sex differences in the severity of hemolytic diseases, such as sickle cell and malaria, as well as the viability of red cell storage and recovery.

**Supplementary Information:**

The online version contains supplementary material available at 10.1186/s12864-022-08461-4.

## Background

Red blood cell (RBC) response to canonical in vitro stressors, such as cold storage, osmotic hemolysis, and oxidative hemolysis, has been associated with altered RBC survival after transfusion [1–3]. In humans, in vitro osmotic stress is a highly reproducible trait [4] that can be further mediated by study donor characteristics such as sex, ancestry, age, donation history, and genetic factors that regulate RBC integrity and functions.

RBC susceptibility to osmotic hemolysis is moderately heritable, with an overall SNP-based heritability of 0.35, which was estimated in the first genome-wide association study (GWAS) for osmotic hemolysis we reported [5]. The most compelling findings of the study were the identification of genes known to modulate RBC structure and function, including *SPTA1* (Spectrin $\mathrm{\alpha }$ chain), *ANK1* (ankyrin 1), *AQP1* (aquaporin 1), *SLC4A1/Band 3* (solute carrier family 4 member 1), and genetic variants in metabolic enzymes *HK1* (hexokinase 1), stress kinases *MAPKAPK5* (Mitogen-Activated Protein Kinase-Activated Protein Kinase 5), ion channels *PIEZO1* (piezo-type mechanosensitive ion channel component 1), and *MYO9B* (myosin IXB). The identified genes have been associated with human RBC disorders such as dehydrated hereditary stomatocytosis [6, 7], spherocytosis [8, 9], ellipto-poikilocytosis [10], xerocytosis [11], and hemolytic anemia [12].

Sex is an influential factor for hemolysis, as males have enhanced susceptibility to hemolysis [1]. The sex dichotomy in RBC susceptibility to hemolysis is present in cold stored RBCs and in hemolytic diseases including sickle cell anemia [13–17]. It is largely unknown if the sex bias is mediated by different genetic mechanisms. In this study, we aimed to identify genetic variants regulating RBC function and hemolysis in a sex-specific manner utilizing data from blood donors in the National Heart, Lung, and Blood Institute RBC-Omics cohort [18]. This diverse, multi-ethnic cohort has 12,231 blood donors with European, African, Hispanic, and Asian ancestry. We evaluated both the sex-stratified GWAS strategy and genome-wide gene-by-sex interaction scan for osmotic analysis during routine blood storage, established the best practice for sex-specific genetic analysis in different scenarios, and characterized the common and different genetic variants regulating RBC hemolysis between male and female sex.

## Results

### Sex-stratified GWAS results for osmotic hemolysis

**Fig. 1:**
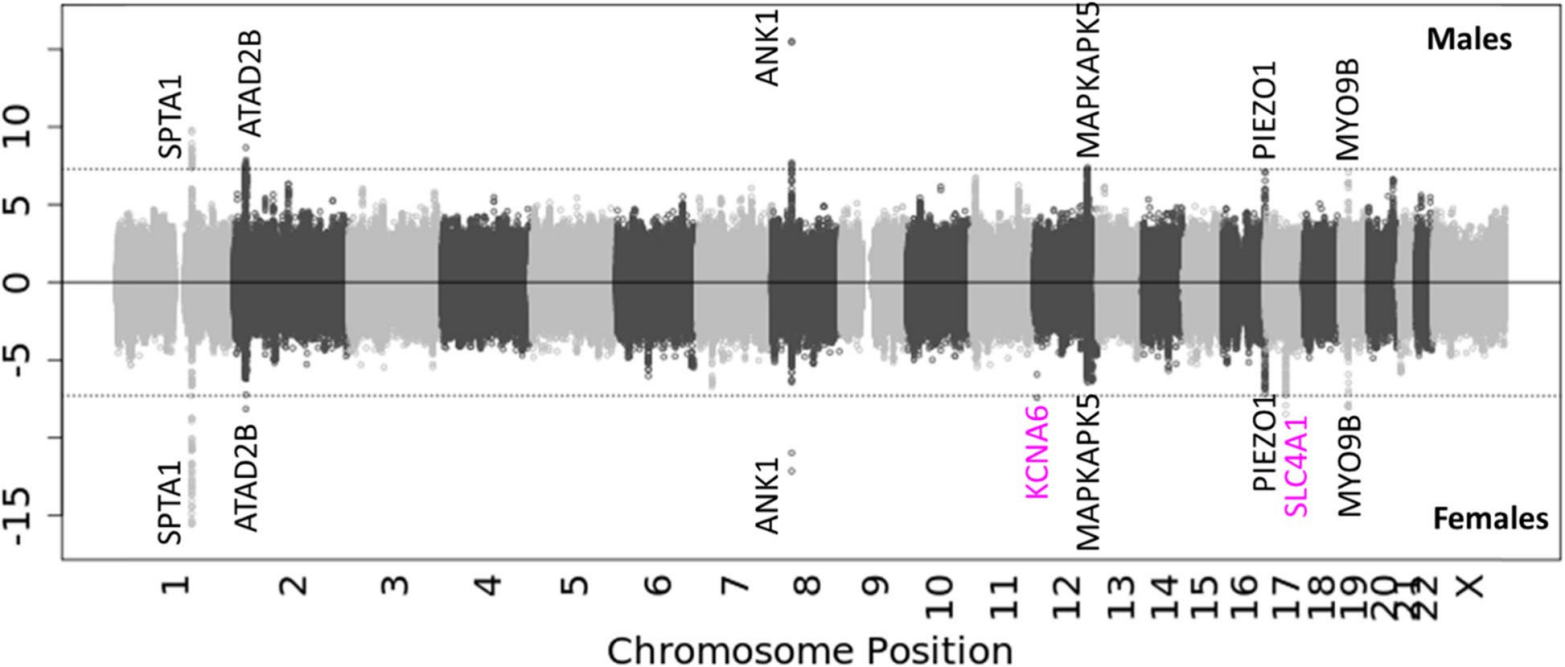
Chicago plot for sex-stratified GWAS for osmotic hemolysis in RBC-Omics. The top panel is for male-specific GWAS, and the bottom panel is for female-specific GWAS. The dotted lines indicate the genome-wide significance ($p<5\times {10}^{-8}$) level. Pink color indicates genome wide significant loci in only female sex. Black gene names are GWA significant in both sexes

We conducted GWAS for osmotic hemolysis stratified by sex. The Chicago plot (Fig. 1) shows that many previously identified loci associated with osmotic hemolysis [5] reached genome-wide significance ($p<5\times {10}^{-8})$ in both sexes: *SPTA1*, *ATAD2B, ANK1*, *MAPKAPK5*, *PIEZO1,* and *MYO9B*. Interestingly, two loci, *KCNA6* and *SLC4A1*, are significantly associated with osmotic hemolysis only in the female sex. QQ plots indicate no systematic bias of the results (Supplementary Fig. S1).

SNP-based heritability was assessed using linkage disequilibrium (LD) score regression [19] in male- and female-specific osmotic hemolysis, respectively. Our results indicated that the heritability for osmotic hemolysis was significantly higher ($p<2\times {10}^{-16}$) in males (${h}_{g}^{2}=0.46, sd=0.12$) than in females (${h}_{g}^{2}=0.41, sd=0.11$).

### Quantified SNP-specific effect size and *p*-value differences derived from sex-stratified GWAS

**Fig. 2:**
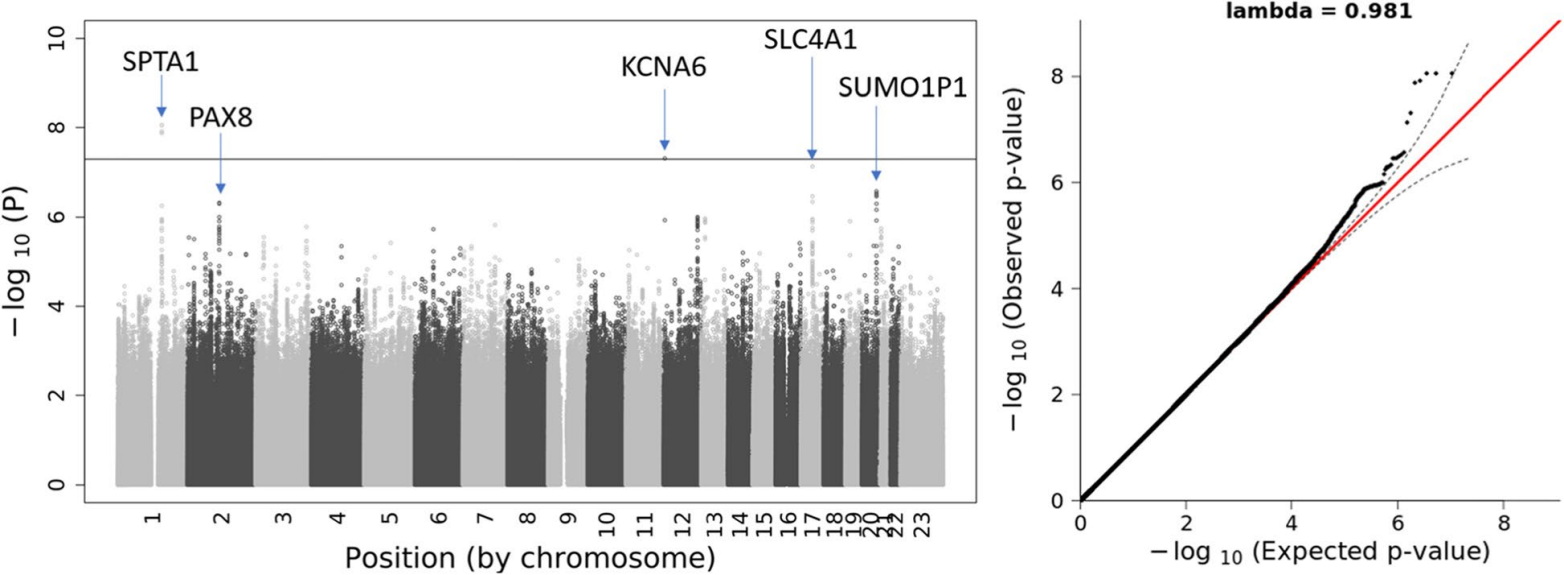
The Manhattan and QQ plot from *p*-value comparison test between male- and female-specific GWAS for osmotic hemolysis in RBC-Omics. The solid line indicates the genome-wide significance ($p<5\times {10}^{-8}$) level

**Fig. 3:**
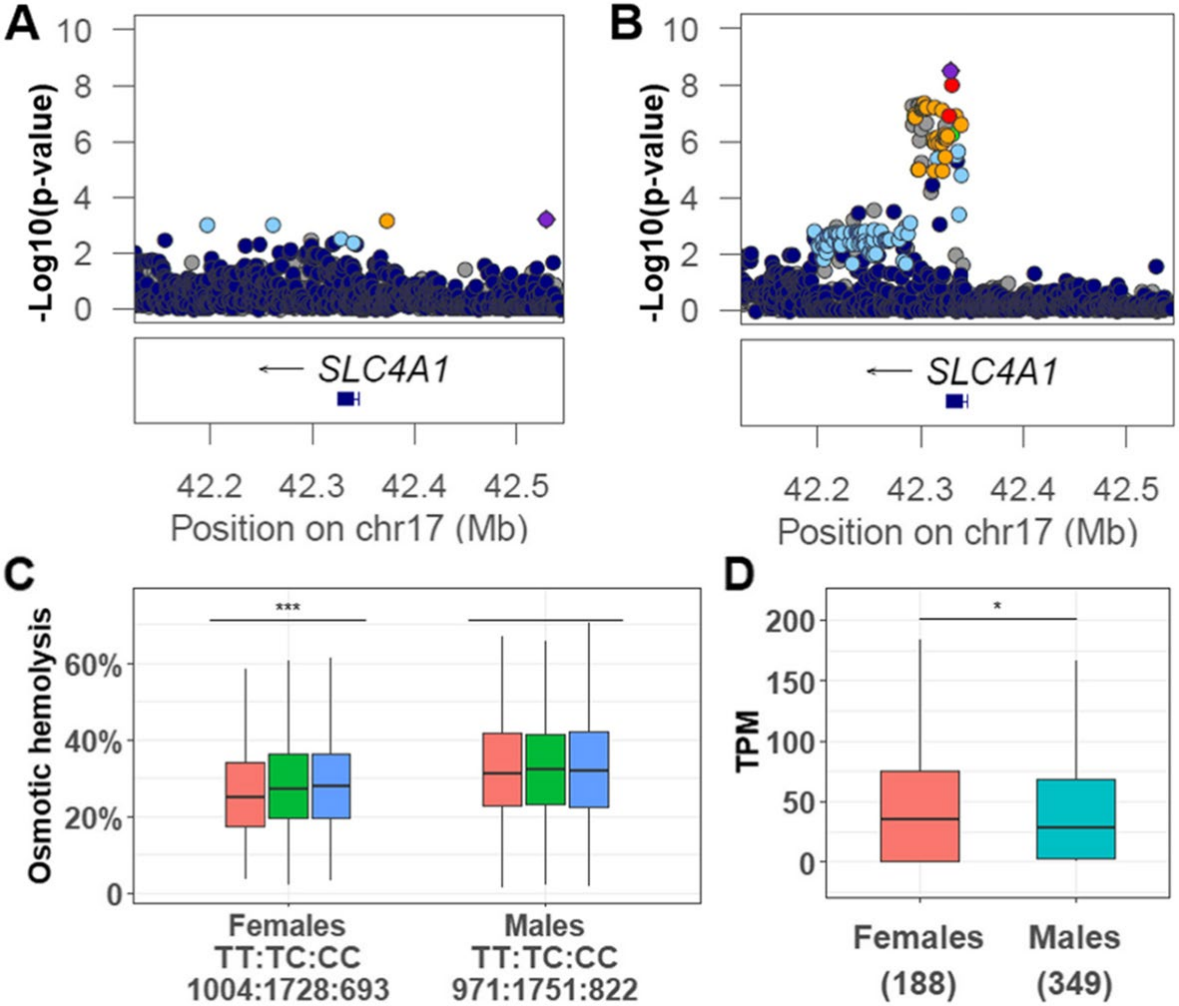
LocusZoom [24] plot for the genomic region around *SLC4A1* from GWAS for osmotic hemolysis (A) in males and (B) in females. (C) SNP rs1476512 within the gene *SLC4A1* is associated with more osmotic hemolysis in females (*p* = 2.57E-05) but not in males (*p* = 0.97). (D) *SLC4A1* shows significantly (*p* = 0.039) more expression (Transcripts Per Million) in whole blood in females than males according to GTEx [25]

Based on the sex-stratified GWAS, we compared the differences between effect sizes and *p*-values at each SNP across the genome. Assuming no relatedness in RBC-Omics data, we first tested the effect size differences between male- and female-specific GWAS (Eq. (2) in Methods). The QQ plot shows no systematic bias, and there is no genome-wide significant difference found for effect sizes (Supplementary Fig. S2). The test for *p*-value difference, based on Eq. (3) in Methods, identified two genome-wide significant ($p<5\times {10}^{-8}$) loci—*SPTA1* and *KCNA6—*and three suggestive ($p<5\times {10}^{-7}$) loci—*PAX8*, *SLC4A1,* and *SUMO1P1* (Table 1, Fig. 2). *SPTA1* is known to modulate RBC function [11], and our results show it has significant effects in both males and females. *KCNA6* is a member of the Potassium Voltage-Gated Channel family, which is involved in cell volume regulation, including RBCs [20]. Results show that genetic variants around *KCNA6* are significantly associated with osmotic hemolysis in females but not in males. The same female-specific associations are observed around the gene *SLC4A1*, which encodes for erythrocyte band 3 protein that plays a pivotal role in regulating anion transport across membranes, and for which mutations are associated with hereditary spherocytosis and the Diego blood group [21–23]. Conversely, genetic variants surrounding the other two genes, *SUMO1P1* (SUMO1 Pseudogene 1), which despite its name is spliced and translated, and *PAX8* (member of the paired box family of transcription factors), demonstrated association with osmotic hemolysis only in males. Taking *SLC4A1* as an example, Fig. 3 illustrates the difference in GWAS results for osmotic hemolysis between males and female and the difference in gene expression in whole blood.

**Table 1:** Top SNPs in comparing the *p*-value differences between sex-stratified GWAS for osmotic hemolysis

rsID	Gene	Chr	A1	A2	AF (A1)	Males (*n* = 6,127)		Females (*n* = 6,102)		*P*-value difference test^b^
						**β-value (A1)**	*P-*value	**β-value (A1)**	***P*-value**	***P*-value**
rs2518489^a^	SPTA1	1	G	A	0.28	1.42	**3.28E-08**	1.93	**2.85E-16**	**8.67E-09**
rs9788072	KCNA6	12	A	G	0.11	0.13	0.74	-2.04	**3.56E-08**	**4.81E-08**
rs13306780^a^	SLC4A1	17	A	C	0.41	0.51	0.043	1.37	**3.14E-09**	**7.28E-08**
rs6068661^a^	SUMO1P1	20	A	G	0.44	-1.27	**2.53E-07**	0.014	0.95	**2.66E-07**
rs11123179	PAX8	2	C	T	0.20	-1.47	**4.77E-07**	0.0028	0.99	**4.81E-07**

^a^These are representatives of other similar SNPs in the same loci

^b^This column shows the *p*-value based on testing the *p*-value difference between male- and female-specific GWAS for osmotic hemolysis

## Genome-wide gene-by-sex interaction scan

We performed a genome-wide gene-by-sex interaction scan using Eq. (4) (see Methods). The joint analysis for SNP main effects and $SNP\times Sex$ interactions indicated an inflation problem (Supplementary Fig. S3, $\lambda =1.55$). Although the results from the joint analysis revealed many consistent loci previously reported to be associated with osmotic hemolysis [5], the global inflation indicates some confounding factors not appropriately controlled in interaction analysis [26], largely because of the diverse, multi-ethnic participants in the RBC-Omics dataset. The top 10 principal components (PCs) accounting for population stratification in regular GWAS are not enough to control the confounding effects in the interaction analysis. To illustrate the influence of the multi-ethnic population structure in the context of interaction analysis, we conducted ancestry-specific gene-by-sex interaction scan in individuals labeled as non-Hispanic White (EUR, *N* = 7,598) and African American (AFR, *N* = 1,036) separately. The genetic ancestry was defined by clustering analysis of the RBC-Omics population overlaid on the 1000 Genome phase 3 samples [5]. The QQ plots for gene-by-sex interaction analyses in both EUR and AFR have no inflation (Supplementary Fig. S4), indicating that the complicated population structure in a multi-ethnic dataset like RBC-Omics was not appropriately controlled using the regular PCs in the interaction model.

## Discussion

This study identified sex-specific genetic determinants of hemolysis that may explain the observed sex dichotomy in RBC susceptibility to osmotic hemolysis of cold stored red cells. We conducted both genome-wide gene-by-sex scan and sex-stratified GWAS for osmotic hemolysis using the REDS-III RBC-Omics cohort. The SNP-based estimated heritability was significantly higher in males than in females (${h}_{g}^{2}=0.46$ vs. ${h}_{g}^{2}=0.41$, $p<2\times {10}^{-16}$).

We have developed a statistic to compare *p*-values from sex-stratified GWAS (Eq. 3). The statistic was used to conduct a genome-wide scan of the genetic variants that quantified differences in the significance of the associations between the sexes. Loci near *KCNA6* and *SLC4A1* were identified as having female-specific associations with osmotic hemolysis. The top SNP at the *SLC4A1* locus, rs13306780, is associated with nine RBC traits according to GWAS Catalog [27]. Our study also identified two male-specific loci associated with osmotic hemolysis, near *SUMO1P1* and *PAX8*. The gene *SUMO1P1* was recently identified as a new member of the small ubiquitin-like modifiers (SUMOs) family, with exceptionally high expression levels in testes and peripheral blood leukocytes [28]. It is involved in the formation and disruption of promyelocytic leukemia-based nuclear structures that regulate various cellular processes. In GWAS Catalog, *SUMO1P1* is associated with RBC traits like mean corpuscular hemoglobin, red cell distribution width, and mean corpuscular volume. The other gene, *PAX8*, is a transcription factor associated with hemoglobin, RBC count, and hematocrit [29, 30]. Interestingly, these genes with sex-specific associations with hemolysis have significant differences ($p<0.05$) in gene expression between males and females in whole blood according to GTEx, except for *KCNA6*, which does not have expression data (Supplementary Table 2). Thus, the validity of the genetic variants identified in this study is supported by the known associations with RBC measurements and the gene expression data in whole blood. These genetic variants likely underlie mechanisms that regulate osmotic hemolysis of RBCs.

One limitation of the statistic developed is that it may identify significant genetic variants in both sexes but to different extents. For example, the genetic variants around *SPTA1* reached genome-wide significance in both male- and female-specific GWAS, with more extreme *p*-values in the female sex. The difference is unlikely caused by detection power, because there were similar sample sizes for both sexes. The expression data for *SPTA1* did not show a difference in whole blood between the sexes. However, in sex-biased eQTL analysis results from GTEx [31], there is one SNP, rs863327, identified as an eQTL for *SPTA1* in whole blood only in females ($p=0.009$), but not in males ($p<0.3$). In our sex-stratified GWAS results, the same SNP, rs863327, was significantly associated with osmotic hemolysis in both males ($p=6.89\times {10}^{-9}$) and females ($p=1.54\times {10}^{-13}$). Therefore, the observed difference in the significance levels of associations may indicate potential variation of the underlying regulation mechanisms between the sexes.

Although the comparison of effect sizes between male- and female-specific GWAS for osmotic hemolysis did not show a significant difference, the tests did reveal genetic variants with opposite effects between the sexes (Supplementary Table 1). The top hit was around the gene *SCFD1* (Sec1 Family Domain Containing 1), which is related to the metabolism of protein pathways. Although the gene expression of *SCFD1* in whole blood did not show a difference between males and females ($p=0.84$), the sex-biased eQTL analysis indicated the existence of sex-specific eQTL for *SCFD1* in whole blood [31].

We performed a genome-wide gene-by-sex interaction scan for osmotic hemolysis using the RBC-Omics data. However, the heterogeneity of ancestry in such a multi-ethnic dataset caused systematic inflation of the *p*-values for the interaction term $SNP\times sex$. To properly control the population stratification in the interaction analysis, covariate-by-gene, covariate-by-sex, PC-by-gene, and PC-by-sex interaction terms should be included in the same model. However, such options are currently limited by available software and heavy computing burden. Recently published work in another multi-ethnic dataset, the Population Architecture using Genomics and Epidemiology study, has demonstrated the benefits and importance of conducting genome-wide genetic analysis in diverse populations to maximize genetic findings and reduce health disparities [32]. Our study raised the question of how to properly control confounding factors when conducting “MEGA” genome-wide interaction analysis in such multi-ethnic datasets. Further development of methods is needed to address the issues, for we believe these confounding effects could hold for any covariate uses for interaction analysis.

Another limitation of the study is the lack of a replication cohort for the in vitro hemolysis measures since RBC-Omics is the first study to explore stress hemolysis as a quantitative trait. Therefore, follow-up studies are needed to validate the findings in the present study.

## Conclusions

In summary, we have assessed sex-specific genetic associations for RBC susceptibility to osmotic hemolysis in RBC-Omics. The ethnically diverse populations in RBC-Omics provide a comprehensive evaluation of the genetic factors but also limit the usage of standard genome-wide gene-by-sex interaction scan method because of the improper control for population stratification in the interaction model. Therefore, we implemented sex-stratified GWAS and then compared both the effect sizes and *p*-values for each genetic variant between the sexes across the genome. The resulted unbiased QQ-plots indicated the validity of the derived statistics. Using this methodology, we found sex heterogeneity for osmotic hemolysis in five loci: *SPTA1*, *KCNA6*, *SLC4A1*, *SUMO1P1,* and *PAX8*. Our results reinforce the need to consider sex-specific associations in characterizing the genetic architecture for sexually dimorphic traits like osmotic hemolysis. Furthermore, the identified loci with sex-specific associations shed light on potential biological mechanisms for understanding the sex differences of osmotic hemolysis with implications for efficacy of RBC transfusions and, more important, relevant to understanding sex differences in penetrance and severity of genetic and acquired hemolytic diseases as well as infectious diseases such as malaria [33–35].

## Methods

### REDS-III RBC-Omics cohort and blood osmotic hemolysis measurement

The REDS-III RBC-Omics study aimed to improve blood transfusion safety by evaluating the association of donor characteristics (e.g., sex, age, race/ethnicity) on blood storage quality and post-transfusion outcomes. The RBC-Omics cohort consists of a multi-ethnic population (12% African American, 12% Asian, 8% Hispanic, 64% white, and 5% multiracial/other) of blood donors with well-characterized demographic, behavioral, and donation history [18]. In total, 13,403 healthy blood donors over the age of 18 were enrolled at four U.S. blood centers. The details for genetic data QC and imputation were described in detail in Page et al. [5]. We removed related samples by keeping one relative per family based on the relatedness estimation using identity-by-descent/identity-by-state (IBD/IBS). For this study, the final informative sample size is 12,231. Table 2 describes the sample characteristics.

**Table 2:** Characteristics of the REDS-III RBC-Omics cohort

Ancestry	No. males	No. female	Male osmotic hemolysis (mean ± SD)	Female osmotic hemolysis (mean ± SD)
White	3,975	3,789	32.88% ± 13.04%	27.73% ± 11.93%
African American	694	773	18.80% ± 10.78%	16.86% ± 10.29%
Asian	807	654	30.40% ± 13.54%	25.18% ± 12.27%
Hispanic	378	568	31.01% ± 12.46%	28.23% ± 12.28%
Other	274	319	30.22% ± 13.65%	24.48% ± 12.66%

As one of the measures indicating blood storage quality, RBC osmotic hemolysis is defined by the loss of hemoglobin in response to reduced osmotic pressure. In REDS-III RBC-Omics, osmotic hemolysis was determined in vitro as the rate of osmotic hemolysis following incubation of washed RBCs (stored for 39–42 days at 1–6 $^\circ C$) in a modified pink test buffer [13], and the measure ranges from 0 to 100%.

Using a multivariable linear model, our previous study [13] has demonstrated that males and older age groups have higher osmotic hemolysis, African American and Asian ethnicity and donation history are negatively associated with osmotic hemolysis. Thus, these modifiers are all included in our models in this study.

### Sex-stratified GWAS

In each sex, linear regression was used to test the association between each SNP and osmotic hemolysis by the software ProbABEL [36]. Models were adjusted for age, donation history, ancestry, and sex-specific top 10 ancestry PCs.

### Approaches to identify differences between sex-stratified GWAS

A general method to detect the differences between the GWAS results stratified by sex is the statistical test for the effect sizes [37]:

1 $$z=\frac{{\beta }_{male}-{\beta }_{female}}{\sqrt{{SE}_{male}^{2}+{SE}_{female}^{2}-2r\bullet {SE}_{male}\bullet {SE}_{female}}}$$

where ${\beta }_{male}$ is the effect size of the genetic variant in male-specific GWAS, and ${SE}_{male}$ is the corresponding standard error. The term $r\bullet {SE}_{male}\bullet {SE}_{female}$ is an estimate of the covariance between ${\beta }_{male}$ and ${\beta }_{female}$, which accounts for the relatedness among samples; $r$ is the Spearman rank correlation coefficient across all SNPs. If assuming no relatedness in the dataset, the test can be simplified to

2 $$z=\frac{{\beta }_{male}-{\beta }_{female}}{\sqrt{{SE}_{male}^{2}+{SE}_{female}^{2}}}$$

In addition to the comparison of the effect sizes, we developed a statistic to compare the *p*-values between sex-stratified GWAS:

3 $$u=\frac{|{p}_{males}-{p}_{females}|}{max({p}_{males},{p}_{females})}\sim U\left[\mathrm{0,1}\right]$$

where ${p}_{males}$ is the *p*-value of the genetic variant in male-specific GWAS. Under the assumption that both ${p}_{males}$ and ${p}_{females}$ follow uniform distribution, the statistic $u$ also follows uniform distribution $U[\mathrm{0,1}]$.

### Genome-wide gene-by-sex interaction scan for the sex difference in osmotic hemolysis

A joint analysis approach simultaneously testing on both SNP main effects and SNP x environment interactions has been employed in gene-environment interaction studies [38–40]. We used the same approach to detect a joint effect of SNP and $SNP\times Sex$ interactions on osmotic hemolysis:

4 $$Y={\beta }_{0}+{\beta }_{SNP}\bullet SNP+{\beta }_{sex}\bullet Sex+{\beta }_{g\times s}\bullet SNP\bullet Sex +{\beta }_{1}\bullet Cov+\varepsilon$$

where $Y$ is the osmotic hemolysis; $Cov$ stands for a set of covariates, such as age, number of donations during the past 2 years, and top 10 PCs accounting for population stratification. The estimation of the coefficients, ${\beta }_{SNP}$, ${\beta }_{g\times s}$ and their covariance matrix can be used to construct a Wald’s statistic, which follows a ${\chi }^{2}$-distribution with two degrees of freedom. The genome-wide gene-by-sex interaction scan was conducted with ProbABEL [36] using the option “–interaction.” Then a customized script calculates Wald’s statistic and corresponding *p*-values based on the output coefficients and covariance estimated.

### SNP-based heritability

Linkage disequilibrium score regression [19] (https://github.com/bulik/ldsc) was used to estimate SNP-based heritability from the GWAS summary statistics, which were filtered by minor allele frequency (MAF > 0.01). The LD scores were precalculated with the 1000 Genome European reference population (https://data.broadinstitute.org/alkesgroup/LDSCORE/eur_w_ld_chr.tar.bz2). To compare the heritability for osmotic hemolysis between males and females, we used t-test based on the estimated heritability, standard deviation, and sample sizes.

## Supplementary Information

**Additional file 1: Figure S1.** The QQ plots from (A) male-specific GWAS for osmotic hemolysis and (B) female-specific GWAS for osmotic hemolysis in RBC-Omics. **Figure S2.** The Manhattan (A) and QQ (B) plots from effect size comparison betweenmale- and female-specific GWAS for osmotic hemolysis in RBC-Omics. **Figure S3.** The Manhattan (A) and QQ (B) plots for the joint analysis of SNP main and interactions for osmotic hemolysis inRBC-Omics, through a Wald’s statistic following a 2-degree freedom -distribution. **Figure S4.** The Manhattan and QQ plots for the joint analysis of SNP main and interactions for osmotic hemolysis inRBC-Omics, through a Wald’s statistic following a 2-degree freedom -distribution,in (A,B) non-Hispanic White individualsand (C,D) African Americans, separately. **Table1.** Top SNPs incomparing the effect sizes between sex-stratified GWAS for osmotic hemolysis. **Table2.** Sex-specific geneexpression data from GTEx[1].

## Data Availability

The individual-level genotype and phenotype data used are all available through dbGap. The dbGap study accession number for REDS-III RBC-Omics data is phs001955.v1.p1 (dbGaP Study (nih.gov)). The codes to run all the analyses can be found at https://github.com/RTIInternational/GeneBySexHemolysis.

## Abbreviations

RBC: Red blood cell
GWAS: Genome-wide association study
MAF: Minor allele frequency
NHLBI: National Heart, Lung, and Blood Institute
REDS: Recipient Epidemiology Donor Evaluation Study
