# Supplementary material for: Sex-specific genetic modifiers identified susceptibility of cold stored red blood cells to osmotic hemolysis
Source: BMC Genomics. 2022 Mar 23;23:227. doi: 10.1186/s12864-022-08461-4 (PMC8941732; doi:10.1186/s12864-022-08461-4)
Supplement: Supplementary file 1 — Additional file 1: Figure S1. The QQ plots from (A) male-specific GWAS for osmotic hemolysis and (B) female-specific GWAS for osmotic hemolysis in RBC-Omics. Figure S2. The Manhattan (A) and QQ (B) plots from effect size comparison betweenmale- and female-specific GWAS for osmotic hemolysis in RBC-Omics. Figure S3. The Manhattan (A) and QQ (B) plots for the joint analysis of SNP main and interactions for osmotic hemolysis inRBC-Omics, through a Wald’s statistic following a 2-degree freedom -distribution. Figure S4. The Manhattan and QQ plots for the joint analysis of SNP main and interactions for osmotic hemolysis inRBC-Omics, through a Wald’s statistic following a 2-degree freedom -distribution,in (A,B) non-Hispanic White individualsand (C,D) African Americans, separately. Table1. Top SNPs incomparing the effect sizes between sex-stratified GWAS for osmotic hemolysis. Table2. Sex-specific geneexpression data from GTEx[1]. [file 12864_2022_8461_MOESM1_ESM.docx]

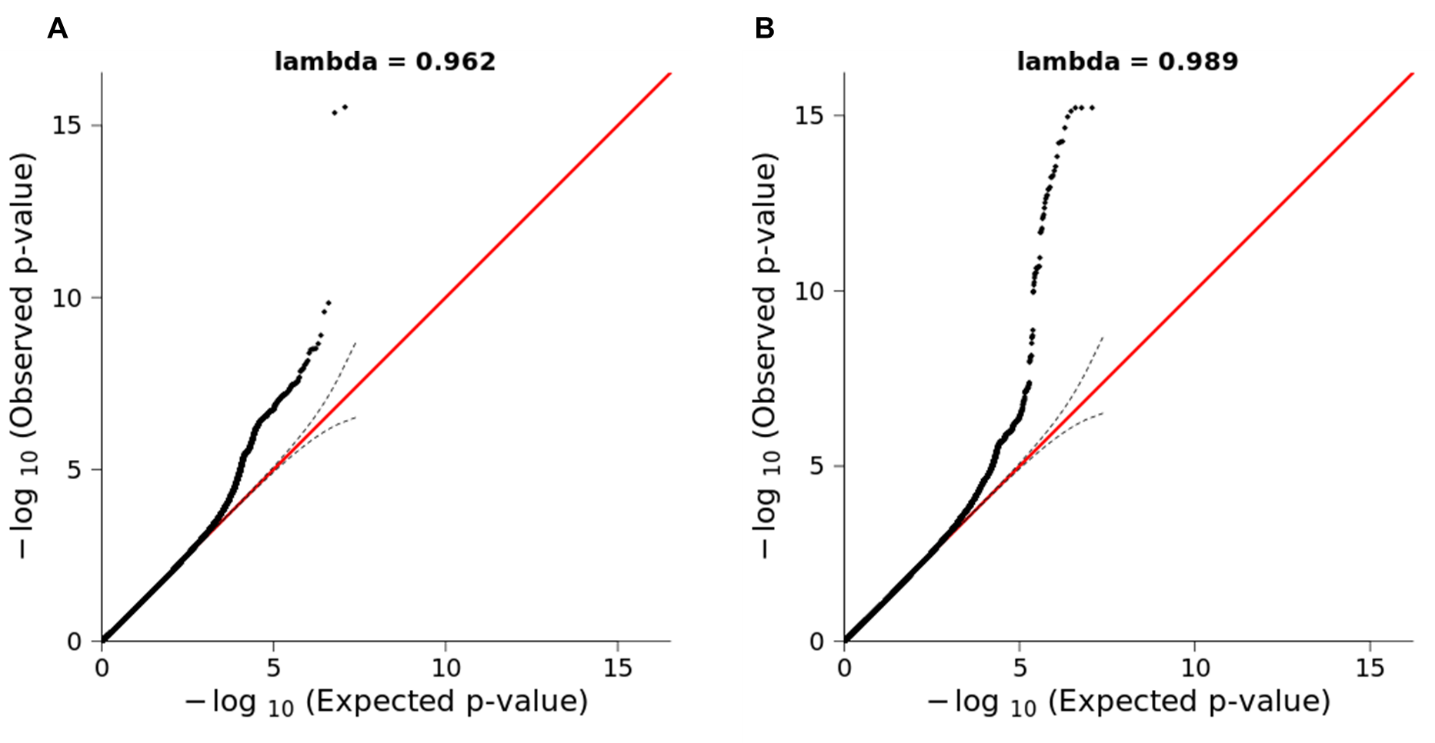


Supplementary Figure S1. The QQ plots from (A) male-specific GWAS for osmotic hemolysis and (B) female-specific GWAS for osmotic hemolysis in RBC-Omics.


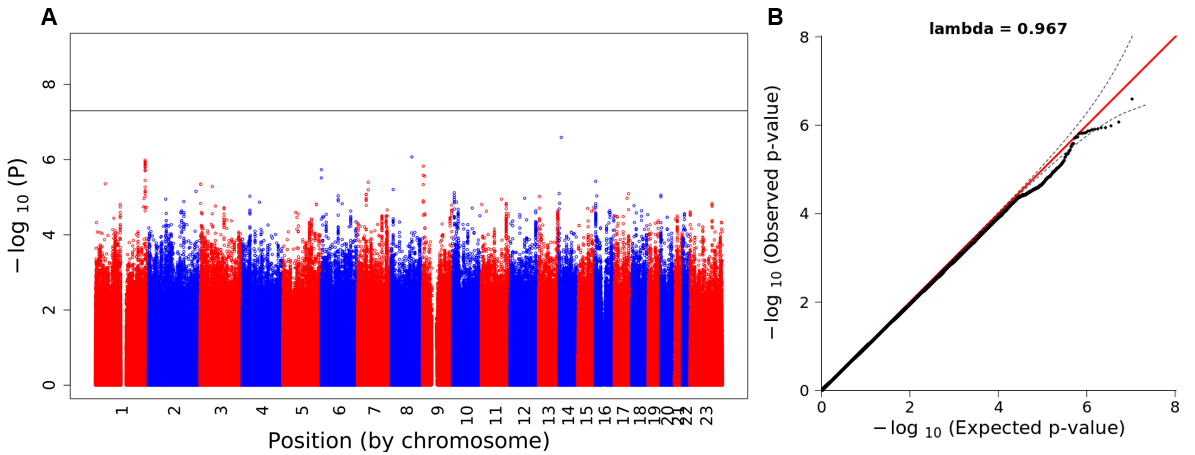


Supplementary Figure S2. The Manhattan (A) and QQ (B) plots from effect size comparison between male- and female-specific GWAS for osmotic hemolysis in RBC-Omics.


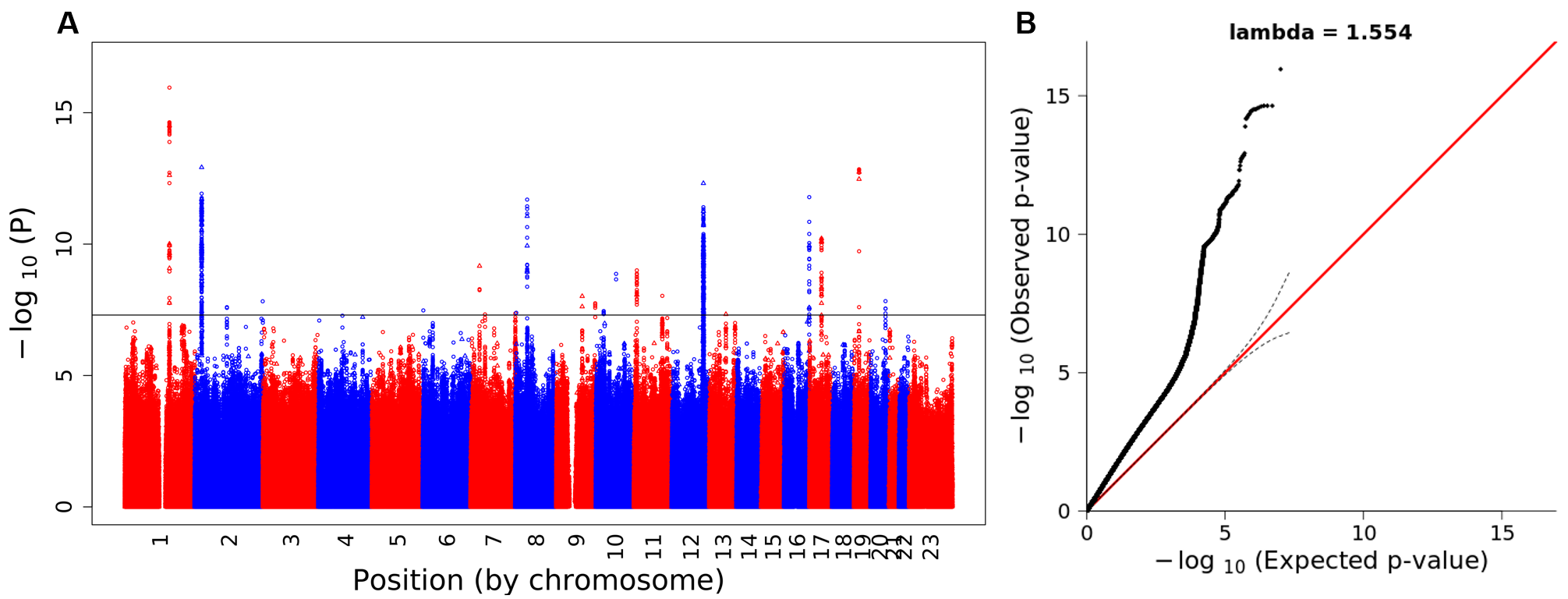


Supplementary Figure S3. The Manhattan (A) and QQ (B) plots for the joint analysis of SNP main and $SNP\times Sex$ interactions for osmotic hemolysis in RBC-Omics, through a Wald’s statistic following a 2-degree freedom $\chi^{2}$-distribution.


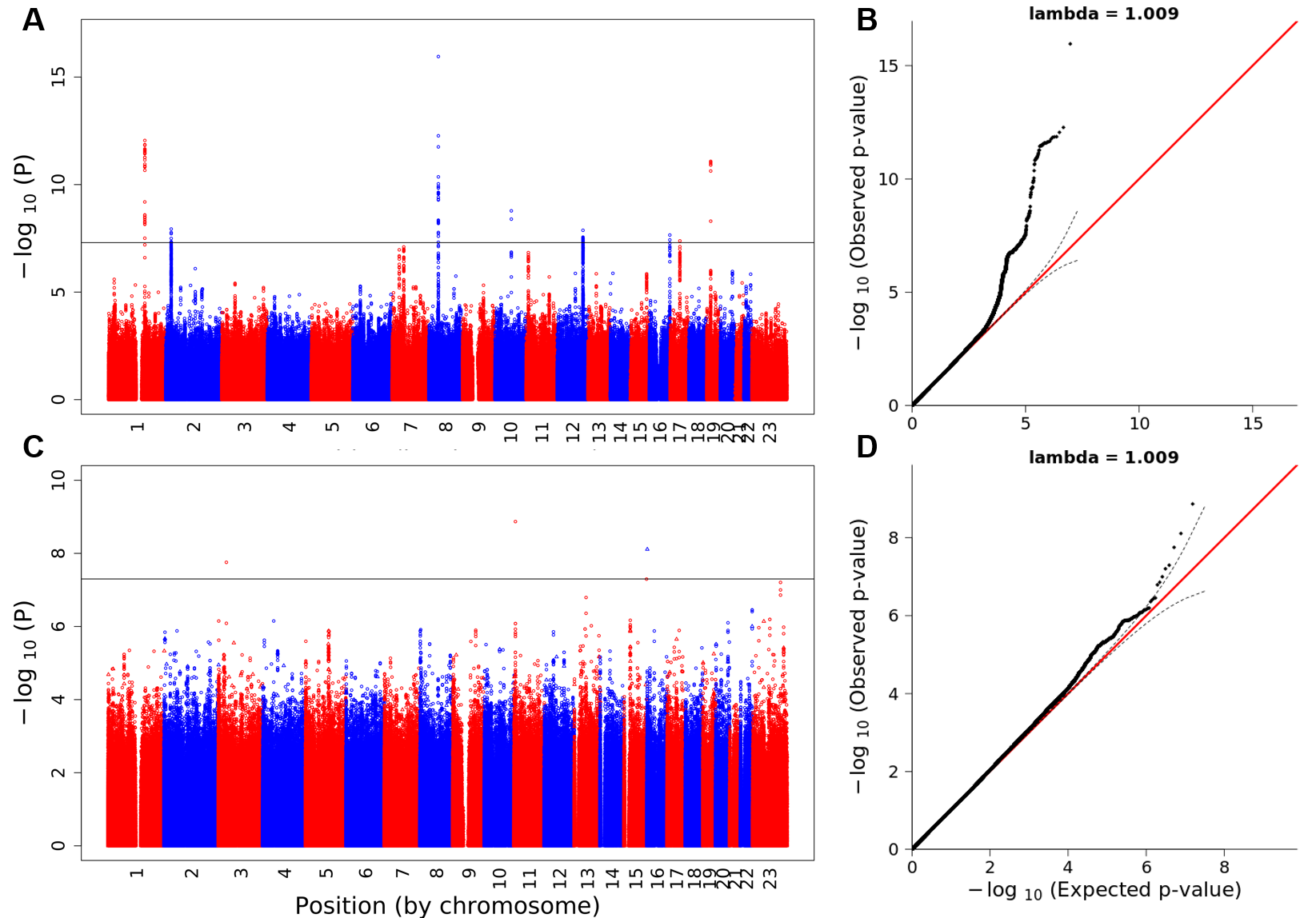


Supplementary Figure S4. The Manhattan and QQ plots for the joint analysis of SNP main and $SNP\times Sex$ interactions for osmotic hemolysis in RBC-Omics, through a Wald’s statistic following a 2-degree freedom $\chi^{2}$-distribution, in (A,B) non-Hispanic White individuals and (C,D) African Americans, separately.

| **Supplementary Table 1. Top SNPs in comparing the effect sizes between sex-stratified GWAS for osmotic hemolysis.** | | | | | | | | | | |
| --- | --- | --- | --- | --- | --- | --- | --- | --- | --- | --- |
| **rsID** | **Gene** | **Chr** | **A1** | **A2** | **AF**  **(A1)** | **Males**  **(n=6127)** | | **Females**  **(n=6102)** | | **Effect size difference test§** |
|  |  |  |  |  |  | **β-value (A1)** | **P-value** | **β-value (A1)** | **P-value** | **P-value** |
| rs75349909 | SCFD1 | 14 | T | C | 0.99 | -4.55 | 4.79E-06 | 2.52 | 0.0077 | 2.57E-07 |
| rs79516434 | C8orf37-AS1 | 8 | G | A | 0.97 | 2.62 | 2.17E-04 | -2.10 | 0.0011 | 8.49E-07 |
| rs58683677* | LINC01354 | 1 | A | C | 0.92 | -1.35 | 0.0022 | 1.56 | 9.84E-05 | 1.03E-06 |
| rs146158994 | GLIS3 | 9 | G | A | 0.91 | 1.60 | 2.61E-04 | -1.22 | 0.0017 | 1.49E-06 |
| rs9378299 | FOXCUT | 6 | G | A | 0.83 | 1.09 | 4.64E-04 | -0.91 | 0.0012 | 1.85E-06 |
| * These are representatives of other similar SNPs in the same loci.  § This column shows the p-value based on testing the effect size difference between male- and female-specific GWAS for osmotic hemolysis. | | | | | | | | | | |

| **Supplementary Table 2. Sex-specific gene expression data from GTEx[1].** | | | | | |
| --- | --- | --- | --- | --- | --- |
| **Gene** | **Males** | | **Females** | | **P-value§** |
|  | N | TPM*  Mean±SD | N | TPM*  Mean±SD |  |
| SPTA1 | 265 | 2.76±5.00 | 142 | 3.99±11.2 | 0.22 |
| KCNA6 | NA | | | | |
| SLC4A1 | 265 | 68.0±74.1 | 142 | 93.5±122.6 | 0.05 |
| SUMO1P1 | 265 | 2.45±2.50 | 142 | 3.02±2.67 | 0.01 |
| PAX8 | 265 | 0.64±0.55 | 142 | 0.56±0.53 | 0.04 |
| SCFD1 | 265 | 9.85±6.00 | 142 | 9.66±5.03 | 0.84 |
| * TPM: Transcripts Per Million.  § This column shows the p-values using Kruskal-Wallis Rank Sum Test. | | | | | |

Reference

1. Consortium GT: **The GTEx Consortium atlas of genetic regulatory effects across human tissues**. *Science* 2020, **369**(6509):1318-1330.
